# From “Homegrown” to Research-Ready: Converting an Existing Practitioner-Developed Violence Prevention Intervention Into an Evaluable Intervention

**DOI:** 10.1177/15248399211031540

**Published:** 2021-08-23

**Authors:** Venita Embry, Rebecca J. Macy, Kathryn E. Moracco, Krista Scheffey, Alexis Moore, Agnieszka McCort, Leah Taraskiewicz

**Affiliations:** 1 University of North Carolina at Chapel Hill, Chapel Hill, NC, USA; 2 RTI International, Research Triangle Park, NC, USA; 3 University of Pennsylvania, Philadelphia, PA, USA

**Keywords:** violence prevention, implementation, program evaluation, community partnerships

## Abstract

There is an increased call for research on promising prevention programs already embedded in communities (“homegrown interventions”). Unfortunately, there is limited guidance to help researchers prepare these types of interventions for rigorous evaluation. To address this need, this article presents our team’s process for revising a promising community-based sexual violence prevention intervention for rigorous research. Our extensive and iterative process of reviewing and revising the intervention was guided by evaluability assessment (EA) approaches, implementation science, and a close collaboration with our community partners. Our EA process allowed us to specify the intervention’s core components and develop a “research ready” standardized curriculum with implementation fidelity assessments. We offer four lessons learned from our process: (1) even with existing materials and an extensive history of community-based delivery, community-developed programs are not necessarily research-ready; (2) close collaboration and a trusting relationship between researchers and community partners throughout the revision process ensures the integrity of core program components are maintained and implementation in diverse community settings is feasible; (3) observations of program implementation are a crucial part of the revision process; and (4) it is important to budget adequate time and resources for such revisions.

Practitioners, researchers, funders, and policy makers concerned with prevention have increasingly called for attention to “practice-based evidence” (Allison et al., 2011; Green, 2006; Knox & Aspy, 2011; Kress et al., 2012; Macy et al., 2017; Serrata et al., 2017). Practice-based evidence is research that evaluates the effectiveness of practices or interventions already embedded in communities that are perceived to be effective but have not been rigorously investigated. Proponents of this paradigm shift argue that focusing on field-developed or “homegrown,” interventions, which have already demonstrated acceptability and feasibility in community-based settings, will make valuable use of limited public health and research dollars, lead to meaningful interventions that are appropriate and sustainable for their contexts, and decrease the lengthy research to practice timeline.

Given the compelling call for practice-based evidence, strategies for enhancing community-developed interventions are needed to ensure that such interventions can be rigorously evaluated. Although enthusiasm for practice-based evidence is growing, little attention has been given to the development of methods to guide collaborative teams of practitioners and researchers in the complex process of conducting community-engaged research and developing practice-based evidence (Begun et al., 2010; Davies & Payne, 2015; Goodman et al., 2018; Nnawulezi et al., 2018; Özdemir & Giannotta, 2014; Ragavan et al., 2019; Secret et al., 2011).

To address this knowledge gap, we describe the process our team of practitioners and researchers used for converting an existing practitioner-developed sexual violence prevention intervention into one that was standardized, research-informed, and evaluable, a process we dubbed going “from homegrown to research ready.” This process was developed in the context of a larger evaluation study, which, in its first phase, aimed to collaboratively identify, refine, and document the components and implementation activities of a violence prevention intervention. This article presents our process, details lessons learned from our practitioner–researcher partnership and offers recommendations for researchers and practitioners tasked with readying community-developed interventions for rigorous evaluation research.

## Background

In the research-based model of intervention development and evaluation, researchers first test the efficacy of interventions within highly controlled environments to establish internal validity, and then assess intervention effectiveness in controlled “real world” conditions using experimental designs and larger samples (Carroll & Nuro, 2002; Fraser & Galinsky, 2010). Although such processes are rigorous, robust, and lead to strong internal validity, demonstrating external validity requires testing in various real-world conditions and settings, which takes considerable time and effort. Thus, research-designed interventions tend to result in a lengthy time gap between research and practice or may not be optimized for real-world implementation in multiple communities (Chorpita, 2002). Researchers and practitioners subsequently need to spend significant time and effort considering how these research-generated interventions are best implemented in practice (Durlak & DuPre, 2008; Fixsen et al., 2009; Özdemir & Giannotta, 2014), including if and how to adapt them to new communities, populations, and settings (Mendel et al., 2008).

Practitioner-developed interventions may have advantages over interventions developed by researchers. First, such “homegrown” interventions are typically developed by practitioners who already work in the intervention’s priority communities and are more likely to attend to local challenges, strengths, and needs (Ragavan et al., 2019). Second, “homegrown” interventions include materials developed in situ, which may improve intervention acceptability and feasibility. In turn, this may save researchers time and resources, since testing the acceptability and implementation of interventions in a variety of contexts is a costly and time-consuming process, which may require separate studies and sources of funding. Finally, promising “homegrown” interventions may already have buy-in from key stakeholders, which is likely to be an essential ingredient for successful intervention sustainment and replication.

While such “homegrown” interventions appear to work well in their communities, research and evaluation are needed to investigate intervention implementation, assess the extent to which an intervention is effective, and promote evidence-based practices for dissemination and wider use. Although community-based service providers are often skilled in developing practical and sustainable interventions, developers and implementers of “homegrown” interventions may lack the capacity to rigorously evaluate their interventions and may not be well positioned to garner competitive external funding for such evaluation (Macy et al., 2017; Secret et al., 2011). Although they may be aware of evidence-based strategies and research studies, practitioners may not have the training and time necessary to conduct rigorous research of their interventions. Thus, in situations where community-based organizations have developed novel “homegrown” interventions, the prevention field can benefit from efforts to evaluate such interventions.

Yet despite the recognized need to test the efficacy and effectiveness of “homegrown” interventions through rigorous research methods, there are few strategies in the extant literature for how to do so. Researchers endeavoring to evaluate community-developed interventions may face significant challenges in conducting studies on interventions that were not originally designed for research. Researchers who are interested in evaluating and establishing “homegrown” interventions as evidence-based require guidance concerning potential processes for readying “homegrown” interventions for research, as these processes are likely to differ from those needed for research-developed interventions. For all these reasons, the goals of this article are to describe our iterative process for understanding and standardizing a “homegrown” intervention for research and provide recommendations for building research–practitioner partnerships and incorporating formative evaluations into practice-based evidence research.

## Approach

### Wise Guys: The Next Level

The community-developed intervention that was the focus of our team’s practice-based research study was Wise Guys: The Next Level (WGNL), which was developed by Children’s Home Society of North Carolina (CHSNC). In its most current practice-based form, WGNL was a 12-chapter, group-based, interactive intervention delivered to young men (i.e., 14–25 years of age) by a prevention educator via 12 weekly sessions, each covering a 60-minute chapter using a manualized curriculum. WGNL aimed to prevent dating and sexual violence perpetration and to increase young men’s knowledge about effective communication, conflict resolution, respectful healthy relationships, and healthy masculinity. CHSNC prevention educators delivered WGNL to groups of young men in diverse community-based and educational settings, such as boys and girls clubs, community colleges, transitional housing programs, residential treatment programs, and sports teams.

WGNL evolved from a program titled Wise Guys, an intervention developed by CHSNC in the 1990s to engage young adolescent males (e.g., typically those in middle school and in the early years of high school) with the topics of healthy masculinity, healthy relationships, and teen pregnancy prevention (Gottsegen & Philliber, 2001; Gruchow & Brown; 2011; Herrman et al., 2016). Recognizing the lack of programming for older adolescent and young men, WGNL was created in 2003 to address similar issues with adolescents and young men aged 14 to 25 years. In recognition of WGNL’s longstanding focus on dating and sexual violence prevention, as well as healthy relationships and positive masculinity, CHSNC was awarded funding to expand its delivery of WGNL by the North Carolina’s Rape Prevention Education Program, which is supported by a Cooperative Agreement with the Centers for Disease Control and Prevention. However, since its development in 2003, WGNL had not been formally evaluated.

Working with the practitioners who developed and implemented WGNL, our team of university-based researchers developed the Guys Relate study to investigate WGNL. The first goal of the evaluation was to collaboratively identify, refine, and document the components and implementation activities of the intervention curriculum. Additional goals were to study WGNL’s promise in preventing sexual violence perpetration and other violence outcomes (e.g., dating and relationship violence). Such a research project was timely and highly relevant because of a myriad studies showing that boys and men are more likely than girls and women to perpetrate sexual violence as well as more severe forms of dating violence, and because limited evidence is available that identifies effective approaches for primary prevention of sexual violence (DeGue et al., 2014; Graham et al., 2019).

Although we anticipated refining the intervention components and activities, on the start of the research project, we realized that additional time and effort were needed to understand and standardize the intervention curriculum for rigorous evaluation. Over the course of 9 months, our research team worked closely with the WGNL developers and implementers at CHSNC to revise, pilot, and standardize the intervention. Importantly, the process used in the development of the “research-ready” WGNL curriculum and intervention was guided and informed by recommended practices in the areas of (1) evaluability assessment and formative research strategies for community-based settings (Leviton et al., 2010; Trevisan & Walser, 2014); (2) a user-centered design approach, which places primary importance on the needs of end users (e.g., both prevention educators who would implement the programs and young men who would participate in the program; Lyon & Koerner, 2016); (3) public health intervention development recommendations to ensure the production of high-quality materials (O’Cathain et al., 2019; Wight et al., 2016); and (4) recommended practices in fidelity instrument and protocol development (Gearing et al., 2011). In the following sections, we detail our processes for preparing the WGNL intervention for research, as well as lessons learned from this process.

### Guys Relate Study

First, our research team realized that we needed a clear and full understanding of the intervention components, including how the curriculum was being delivered and under what conditions (e.g., setting, type of participants, number of chapters covered per week, length of each delivered chapter). Working closely with CHSNC, we discovered that, as with many “homegrown” interventions, the existing WGNL curriculum was not standardized in its implementation. For example, the WGNL curriculum was more than 300 pages long and contained more content than could feasibly be delivered in twelve 60-minute sessions. As such, there was variability in delivery, based on the prevention educators’ discretion and individualized assessments of the appropriateness of specific curriculum content and activities for the setting (e.g., younger versus older adolescent participants). Additionally, there was no guidance that identified the intervention’s core components or essential content. Though some flexibility is reasonable and desirable, to evaluate WGNL we required greater consistency in intervention content and delivery.

**Figure 1:**
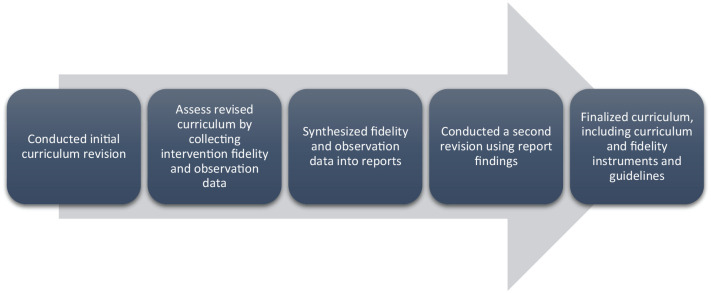
Overall Process for Converting an Existing Practitioner-Developed Violence Prevention Intervention Into an Evaluable Intervention

Thus, our first goal was to develop a standardized, “research-ready” curriculum for WGNL to stand up for an evaluation study. However, we also wanted to maintain the essence of the existing WGNL curriculum and evaluate an intervention that was relatively adaptable for diverse audiences and settings. In addition, as outsiders to the WGNL intervention, we did not want to make determinations about what the core components of the intervention were, nor the best mode of delivery. Instead, we undertook an iterative process of reviewing and revising the intervention in active and close collaboration with our community partners at CHSNC. This process is depicted in Figure 1. By working closely with the program implementers, we sought to ensure that any revisions made to the intervention were consistent with the developers’ aims and intentions.

### Initial WGNL Curriculum Revision

Our research team and community partners reviewed the existing WGNL curriculum to assess its content and prominent constructs. A major challenge in this initial revision was managing the size of the existing WGNL curriculum, which included 12 chapters (implemented over 12 weekly “sessions”), each with five to 11 activities. In any given session, the prevention educators selected their preferred activities from the chapters, based on their familiarity with and preferences for the materials, as well as what they thought would work best with current participants. To make the intervention research-ready, as a key part of this initial revision, we aimed to reduce the length of the curriculum to one that could be delivered with fidelity across diverse Guys Relate study sites. Collaboratively, our researcher and practitioner team worked to identify the core content and activities for each of the 12 chapters in the original curriculum that would be included in the standardized intervention. We also decided to include no more than three activities per chapter. Thus, for each of the 12 chapters, three WGNL activities were chosen by the implementers as those that best aligned with the specific chapter objectives, larger curriculum objectives, and had previously demonstrated high participant engagement in practice.

In addition, we used this initial revision process to (1) ensure that the curriculum and intervention was GLBTQ+ inclusive (e.g., adding same-gender couple scenarios among other strategies); (2) update the curriculum content to reflect the lives of contemporary adolescents and young adults (e.g., including the participants’ use of mobile phones for texting and incorporating social media in the WGNL content and activities); and (3) update the curriculum content to reflect the most current evidence and knowledge concerning the intervention’s key topics of healthy relationships, positive masculinities, and dating and sexual violence prevention. During this initial revision, we also developed detailed chapter-specific fidelity instruments to be completed after each chapter by the prevention educators. Complementary, chapter-specific observation forms were also completed by our research team.

### Intervention and Evaluability Assessment of the First Revised WGNL Curriculum

Evaluability assessments is a systematic process that can determine if the program activities are feasible, clearly defined, align with objectives, can be carried out consistently as planned, and have defined resources and processes that can reasonably lead to a successful outcome evaluation. Evaluability assessments can help researchers identify challenges to implementation, areas for revision or clarification. During the WGNL evaluability assessment, the prevention educators delivered the revised curriculum to two separate groups of young men, each at different community-based youth organizations, over the course of 12 weeks. We assessed the implementation and evaluability of the revised curriculum using prevention educators’ self-report of implementation fidelity, as well as research team members’ structured observations of intervention delivery in two settings.

In the chapter-specific fidelity logs, program implementers documented and responded to detailed questions about their decision-making process for any adaptations, additions, or removal of chapter content. Similarly, in complementary observation forms, external observers noted what modifications they observed, as well as information about the context in which they occurred. Each of the fidelity logs and observation forms were three to five pages long and developed using fillable pdf forms. We encouraged the implementers and observers to note contextual information about implementation that would be helpful for the larger team to discuss (e.g., site location challenges, participation, implementer delivery style, and participant responsiveness).

### Synthesized Fidelity and Observation Data

The fidelity logs and observation forms were completed by the implementers and observers, respectively, and submitted to the research team within 48 hours of delivery. Members of our research team analyzed the data in the completed fidelity tools and developed chapter-specific implementation reports that described the actual implementation of each WGNL chapter. By analyzing these data, we were able to assess the degree to which the revised curriculum covered the core content and learning objectives of WGNL chapters, areas of the intervention that could be further improved, and participants’ reactions to the revised curriculum.

This process resulted in 12 unique reports that summarized the implementation fidelity and observation data for WGNL delivery in practice and across two community-based intervention sites for all 12 WGNL chapters. These reports included findings summarizing what aspects of the curriculum were delivered as intended, what was not delivered as intended, other notes, and follow-up questions for either the implementer, the observer, or the larger research team. After a draft report was created for each chapter, we shared it with the educator and observer for their review and comments. When necessary, reports were revised and updated based on the educators’ and observers’ feedback.

The intervention delivery summary reports formed the basis of a second round of curriculum revisions. These reports helped us identify unresolved intervention challenges and potential problems, as well as promising innovations and intervention improvements. For example, we discovered that the educators enhanced curriculum activities and content in ways that strengthened intervention delivery but had not yet been documented in the curriculum manual. We learned that the prevention educators had key activities for each chapter that were frequently and consistently implemented. We also noted that some curriculum activities and content were not essential nor typically addressed in practice and could be removed from the extant curriculum.

### Second WGNL Revision

Guided by the chapter-specific reports, over the course of approximately 3 months and aided by a series of meetings and discussions between our research team and community-based partners, we conducted a second revision of the WGNL curriculum.

In this second round of revisions, we eliminated WGNL content and activities that were not being regularly and fully implemented in practice. We adjusted the sequence of the chapters in order to present foundational topics, such as communication skills and gender norms, before tackling the more complex issues, such as consent and unhealthy and abusive relationships. Ultimately, this extensive revision process, which was guided by findings from the fidelity and observation data, resulted in a substantially shorter curriculum composed of nine chapters, each with two to three key activities. In addition, we used this round of revisions as an opportunity to standardize the formatting of the chapters, content, and activities across the curriculum manual, as well as to revise instructions to ensure that each of the nine chapters could be feasibly delivered in the same way, in the same order, in 60 minutes or fewer. Collectively, all the efforts to streamline the program and its implementation resulted in a curriculum manual that reflected the program as it was delivered in reality and would take less time and fewer resources to implement.

At the end of this second round of revisions, we developed a revised WGNL curriculum, which included (1) chapter-specific fidelity protocols that prevention educators could use to guide program implementation, (2) fidelity logs that educators and Guys Relate researchers could use to document and assess intervention implementation, and (3) observation logs that researchers could use during study observations of the intervention’s delivery to gather implementation data to complement the fidelity logs. These final products, which would ensure consistency of the intervention’s content and delivery, were then ready for use in future studies.

In practice, for each of the intervention’s chapters, the revised fidelity and observation logs assessed various dimensions of intervention fidelity, including (1) whether and what content is delivered, (2) how and to what extent content is delivered, (3) educators’ and researchers’ reflections on any necessary interventions adaptations, and (4) educators’ and researchers’ assessments of participant engagement. Specifically, the logs’ items captured: (1) total number of participants in attendance, (2) how many participants were presented for at least half of delivered chapter, (3) length of chapter delivery, (4) any interruptions to chapter delivery, (5) adherence to chapter key terms defined, (6) completed activity content, (7) how activities were delivered, (8) reasons for any adaptations in any activities and/or content, (9) participant responsiveness, and (10) ideas for implementation improvements. To sum, the structured logs help promote the delivery and documentation of the core activities and content while also enabling educators and researchers to note common adaptations and to record implementation changes in easy ways (Kimber et al., 2019; Kutash et al., 2012).

### Lessons Learned, Implications, and Recommendations

As noted earlier, in the peer-reviewed literature, limited attention has been given to the development of methods and strategies to guide collaborative teams of practitioners and researchers in the complex process of conducting community-engaged research and developing practice-based evidence. As shown in Table 1, we highlight a few key lessons learned from our practitioner–researcher partnership to ready a “homegrown” intervention for research with the goal informing future research and practice. These recommendations may also be helpful for practitioners and/or researchers as they plan their own projects, consider project budgets and timelines, and develop research activities.

**Table 1:** Key Lessons Learned and Recommendations for Converting a Practitioner-Developed Violence Prevention Intervention into an Evaluable Intervention

Lesson learned	Recommendations
Consider to what extent a practice-developed program is research-ready	• Conduct an evaluability assessment to investigate (a) the feasibility of delivering the intervention consistently and with fidelity in the context of a research study and (b) whether the intervention is evaluable in its current form. • With the community partners, determine and confirm the core components of the intervention. • Use the evaluability assessment to distinguish between intervention components from implementation delivery characteristics.
Foster a close collaboration with community partners	• Develop and foster a positive, collaborative relationship with community partners from project design and maintain through project end. • Include community partners as active members of the team (e.g., attend meetings, review program evaluation materials and protocols). • Consult with community partners frequently to determine if revisions keep the core components of the intervention intact and is feasible for implementation. • Present evaluation plan and data collection materials to practice partners to invite their feedback, insights, and reactions, and then revise as necessary.
Observations of intervention implementation are essential	• Ensure that practitioners and researchers have a similar understanding of and agree on key research activities and strategies (e.g., adaptations, fidelity, implementation, observations). • Create a systematic intervention revision plan that includes the collection of various data sources (e.g., data concerning fidelity and implementation from both practitioners and researchers, structured observations, facilitator interviewers) to inform intervention revisions in preparation for future research. • Conduct observations to gain insights in typical implementation and understand what revisions are needed that are both feasible and relevant to the intervention. • Compile findings from the various data sources into short reports that can, in turn, guide intervention refinements and revisions.
Budget adequate time and resources for evaluability assessment and intervention revisions	• To accommodate the time and labor-intensive process of getting a program research-ready, allocate sufficient project time, funding, and other resources. • Plan to incorporate the time and effort of community partners in the evaluability assessment and revisions process.

#### Consider to What Extent a Practice-Developed Program Is Research-Ready

At the beginning, we knew that WGNL was a curriculum that had been successfully delivered in the field for over a decade. Given its long-standing and successful implementation, our practitioner and research team initially assumed that only minor changes would be needed to make the intervention evaluable. However, as we began to develop the study design and implementation plan, it became apparent that a substantial level of revision to the curriculum was needed to ensure a successful evaluation. Specifically, it was not feasible to deliver the entire 300+ pages of the curriculum over the proposed 12-week intervention period. Moreover, in practice, the community partners were not delivering the entire curriculum. As seen in our process, evaluability assessment can help streamline and standardize “homegrown” interventions for evaluation, broader dissemination, and identifying implementation challenges for researchers.

In practice, interventions are often adapted to the needs of the intended population (Cohen et al., 2008). Consequently, over time the implemented intervention may drift from the initial intervention model. Throughout an evaluability assessment, researchers and community partners must come to the table to explicitly identify the core components of the intervention and levels of acceptable flexibility in delivery. As opposed to researchers, those community partners implementing “homegrown” programs have more insight in to “what works” in practice and can advise the researchers about potential areas for flexibility in intervention delivery. Thus, when evaluating a “homegrown” intervention, evaluability assessments are recommended to examine the feasibility of delivering the intervention as instructed in the intervention materials—if they exist—and whether the intervention is evaluable in its current form. Via an evaluability assessment, such insights concerning intervention flexibility from community partners can be incorporated into the intervention design from the beginning, rather than being considered a liability for research.

#### Foster a Close Collaboration With Community Partners

Although this recommendation is one that is mentioned frequently for community-engaged and -based research, it is worth underscoring here. Our team quickly learned that one of the greatest benefits to our project was having a close working relationship and mutual trust with the intervention developers and including them as active members of the study team as funded partners. Community partners can ensure that revisions keep the core components of a “homegrown” intervention intact and that the proposed revisions are appropriate for the context and do not introduce new implementation challenges. Specifically, insights from these community partners who implement WGNL were essential to our understanding of the intervention’s delivery, how participants respond to specific activities or concepts, and how to overcome implementation challenges. In addition, by providing funding for our community partners’ work on the project, their time was supported to attend meetings as well as to provide input and reviews of all revisions to intervention materials (e.g., theoretical framework, changes to curriculum, and fidelity measures). By developing a strong, active, transparent partnership with our community partners, work to revise the curriculum into a research-ready intervention became a shared effort. Moreover, every revision to the curriculum was endorsed by the partners and, in many cases, our research team relied on these partners to make final decisions about the intervention.

Furthermore, collaborative evaluability assessments can help build research capacity among community partners for the development and implementation of evidence-based strategies. Though practitioners are often experts at develop and implementing programs in their communities, they may lack knowledge, resources, and skills for conducting a rigorous evaluation. Through an evaluability assessment project with researchers, practitioners can develop and strengthen their own evaluation knowledge and skills, which in turn can help guide their future efforts in program development, evaluation, and implementation, even after the partnership with researchers ends.

#### Observations of Intervention Implementation Are Essential

Observing the intervention as implemented in the field was a crucial part of our revision process. The observation findings gave our research team much needed insights about implementation in practice settings as well as important information about necessary revisions to help ensure the feasibility and relevance of the intervention. Moreover, the observation process helped our research team develop a common language with our community partners (e.g., defining a chapter objective), as well as identify and clarify concepts that were ambiguous or highly variant during delivery. In all, the observations helped our research team make reasonable adjustments to the intervention to ensure its evaluability and helped us develop fidelity assessment tools that were meaningful for the intervention as it was actually being delivered.

We encourage future, similar research efforts to consider adopting an observation strategy as formative research that is built into the study timeline, along with time for incorporating any needed revisions. Specifically, researchers who are evaluating community-developed interventions should incorporate, in early project stages, an evaluability assessment plan that incorporates routine assessments of current implementation delivery, a clear yet flexible revision plan developed with practitioners, and a collaborative development of process and outcome evaluation plans.

#### Budget Adequate Time and Resources for Evaluability Assessment and Intervention Revision

Getting a practice-based program ready for research can be time and labor-intensive. Our experience involved an extensive, iterative process of revising the existing curriculum and developing and refining fidelity assessments while maintaining the core elements of the original WGNL curriculum. Notably, the entire revision process took over 9 months. Thus, we encourage other research teams to anticipate substantial time devoted to evaluability assessment and revisions, and to allocate sufficient time, funding, and other resources to undertake such efforts, including supporting the time and effort of community partners.

## Conclusion

This article addresses the endeavor of preparing existing community-based interventions for rigorous process and outcome evaluation by describing our process, challenges, and lessons learned converting a “homegrown” sexual violence prevention intervention into one that is “research ready.” Though community-developed interventions may be manualized and involve practitioners who already work with the intervention’s priority communities and are more aware of local challenges, strengths, and needs (Ragavan et al., 2019), researchers must anticipate and plan for a different set of evaluation challenges than what may be expected for researcher-developed interventions. However, there are limited guidance for researchers and community partners engaging in this endeavor.

Researchers interested in engaging in a similar process are strongly encouraged to conduct an evaluability assessment to determine whether the “homegrown” intervention in its current form can be implemented consistently and with fidelity. As an evaluability assessment may require multiple site visits (or structured observations), revising program materials, and interviews with practitioners, it is important for researchers to budget adequate time and effort to thoroughly understand the intervention. Funders should recognize the importance of evaluability assessment and other forms of formative assessments to bridge the gap between practice and research and adequately support these efforts as part of their funding awards.

Finally, we encourage other research-practitioner teams to document and disseminate their strategies and processes for converting “homegrown” into “research ready” interventions. The extant literature on prevention interventions is dominated by traditional researcher-developed and evaluated interventions rather than interventions developed by community-based practitioners who possess insider knowledge about the appropriateness and feasibility of prevention efforts.

This article presents our process, lessons learned, and recommendations for converting community-developed interventions for rigorous evaluation research, filling an essential gap in the literature for advancing evidence-based health promotion programs and interventions. By developing strategies to produce “practice-based evidence,” we may increase the probability that prevention interventions are consistently implemented and effective in real-world contexts.

## References

[bibr1-15248399211031540] Allison K. W. Edmonds T. Wilson K. Pope M. Farrell A. D. (2011). Connecting youth violence prevention, positive youth development, and community mobilization. American Journal of Community Psychology, 48(1–2), 8–20. 10.1007/s10464-010-9407-9 21246272

[bibr2-15248399211031540] Begun A. L. Berger L. K. Otto-Salaj L. L. Rose S. J. (2010). Developing effective social work university-community research collaborations. Social Work, 55(1), 54–62. 10.1093/sw/55.1.54 20069941

[bibr3-15248399211031540] Carroll K. M. Nuro K. F. (2002). One size cannot fit all: A stage model for psychotherapy manual development. Clinical Psychology: Science and Practice, 9(4), 396–406. 10.1093/clipsy.9.4.396

[bibr4-15248399211031540] Chorpita B. F. (2002). Treatment manuals for the real world: Where do we build them? Clinical Psychology: Science and Practice, 9(4), 431–433. 10.1093/clipsy.9.4.431

[bibr5-15248399211031540] Cohen D. J. Crabtree B. F. Etz R. S. Balasubramanian B. A. Donahue K. E. Leviton L. C. Clark E. C. Isaacson N. C. Stange K. C. Green L. W. (2008). Fidelity versus flexibility: Translating evidence-based research into practice. American Journal of Preventive Medicine, 35(5), S381–S389. 10.1016/j.amepre.2008.08.005 18929985

[bibr6-15248399211031540] Davies R. Payne L. (2015). Evaluability assessments: Reflections on a review of the literature. Evaluation, 21(2), 216–231. 10.1177/1356389015577465

[bibr7-15248399211031540] DeGue S. Valle L. A. Holt M. K. Massetti G. M. Matjasko J. L. Tharp A. T. (2014). A systematic review of primary prevention strategies for sexual violence perpetration. Aggression and Violent Behavior, 19(4), 346–362. 10.1016/j.avb.2014.05.004 29606897 PMC5875446

[bibr8-15248399211031540] Durlak J. A. DuPre E. P. (2008). Implementation matters: A review of research on the influence of implementation on program outcomes and the factors affecting implementation. American Journal of Community Psychology, 41(3–4), 327–350. 10.1007/s10464-008-9165-0 18322790

[bibr9-15248399211031540] Fixsen D. L. Blase K. A. Naoom S. F. Wallace F. (2009). Core implementation components. Research on Social Work Practice, 19(5), 531–540. 10.1177/1049731509335549

[bibr10-15248399211031540] Fraser M. W. Galinsky M. J. (2010). Steps in intervention research: Designing and developing social programs. Research on Social Work Practice, 20(5), 459–466. 10.1177/1049731509358424

[bibr11-15248399211031540] Gearing R. E. El-Bassel N. Ghesquiere A. Baldwin S. Gillies J. Ngeow E. (2011). Major ingredients of fidelity: A review and scientific guide to improving quality of intervention research implementation. Clinical Psychology Review, 31(1), 79–88. 10.1016/j.cpr.2010.09.007 21130938

[bibr12-15248399211031540] Goodman L. A. Thomas K. A. Nnawulezi N. Lippy C. Serrata J. V. Ghanbarpour S. Sullivan C. Bair-Merritt M. H. (2018). Bringing community based participatory research to domestic violence scholarship: An online toolkit. Journal of Family Violence, 33(2), 103–107. 10.1007/s10896-017-9944-1

[bibr13-15248399211031540] Gottsegen E. Philliber W. W. (2001). Impact of a sexual responsibility program on young males. Adolescence, 36(143), 427–433. 11817625

[bibr14-15248399211031540] Graham L. M. Embry V. Young B. R. Macy R. J. Moracco K. E. Reyes H. L. M. Martin S. L. (2019). Evaluations of prevention programs for sexual, dating, and intimate partner violence for boys and men: A systematic review. Trauma, Violence, & Abuse, 22(3), 439–465. 10.1177/1524838019851158 31262233

[bibr15-15248399211031540] Green L. W. (2006). Public health asks of systems science: To advance our evidence-based practice, can you help us get more practice-based evidence? American Journal of Public Health, 96(3), 406–409. 10.2105/AJPH.2005.066035 16449580 PMC1470512

[bibr16-15248399211031540] Gruchow H. W. Brown R. K. (2011). Evaluation of the Wise Guys male responsibility curriculum: Participant-control comparisons. Journal of School Health, 81(3), 152–158. 10.1111/j.1746-1561.2010.00574.x 21332480

[bibr17-15248399211031540] Herrman J. W. Moore C. Rahmer B. (2016). Focus on teen men: Evaluating the effectiveness of the Wise Guys program. Journal of Child and Adolescent Psychiatric Nursing, 29(1), 37–43. 10.1111/jcap.12133 26991126

[bibr18-15248399211031540] Kimber M. Barac R. Barwick M. (2019). Monitoring fidelity to an evidence-based treatment: Practitioner perspectives. Clinical Social Work Journal, 47(2), 207–221. 10.1007/s10615-017-0639-0

[bibr19-15248399211031540] Knox L. M. Aspy C. B. (2011). Quality improvement as a tool for translating evidence-based interventions into practice: What the youth violence prevention community can learn from healthcare. American Journal of Community Psychology, 48(1–2), 56–64. 10.1007/s10464-010-9406-x 21267776

[bibr20-15248399211031540] Kress H. C. Noonan R. Freire K. Marr A. Olson A. (2012). Top 20 violence and injury practice innovations since 1992. Journal of Safety Research, 43(4), 257–263. 10.1016/j.jsr.2012.08.002 23127674 PMC11521115

[bibr21-15248399211031540] Kutash K. Cross B. Madias A. Duchnowski A. J. Green A. L. (2012). Description of a fidelity implementation system: An example from a community-based children’s mental health program. Journal of Child and Family Studies, 21(6), 1028–1040. 10.1007/s10826-012-9565-5

[bibr22-15248399211031540] Leviton L. C. Khan L. K. Rog D. Dawkins N. Cotton D. (2010). Evaluability assessment to improve public health policies, programs, and practices. Annual Review of Public Health, 31, 213–233. 10.1146/annurev.publhealth.012809.103625 20235852

[bibr23-15248399211031540] Lyon A. R. Koerner K. (2016). User-centered design for psychosocial intervention development and implementation. Clinical Psychology: Science and Practice, 23(2), 180–200. 10.1111/cpsp.12154 29456295 PMC5812700

[bibr24-15248399211031540] Macy R. J. Ermentrout D. M. Redmond P. H. Jr. Rizo C. F. Pollock M. D. (2017). From novel to empirical: Developing community-based programs into research-ready programs. Child Welfare Journal, 94(3), 151–166.

[bibr25-15248399211031540] Mendel P. Meredith L. S. Schoenbaum M. Sherbourne C. D. Wells K. B. (2008). Interventions in organizational and community context: A framework for building evidence on dissemination and implementation in health services research. Administration and Policy in Mental Health and Mental Health Services Research, 35(1–2), 21–37. 10.1007/s10488-007-0144-9 17990095 PMC3582701

[bibr26-15248399211031540] Nnawulezi N. Lippy C. Serrata J. Rodriguez R. (2018). Doing equitable work in inequitable conditions: An introduction to a special issue on transformative research methods in gender-based violence. Journal of Family Violence, 33(8), 507–513. 10.1007/s10896-018-9998-8

[bibr27-15248399211031540] O’Cathain A. Croot L. Sworn K. Duncan E. Rousseau N. Turner K. Yardley L. Hoddinott P. (2019). Taxonomy of approaches to developing interventions to improve health: A systematic methods overview. Pilot and Feasibility Studies, 5(1), 41. 10.1186/s40814-019-0425-6 30923626 PMC6419435

[bibr28-15248399211031540] Özdemir M. Giannotta F. (2014). Improving dissemination of evidence-based programs through researcher–practitioner collaboration. New Directions for Youth Development, 2014(141), 107–116. 10.1002/yd.20090 24753282

[bibr29-15248399211031540] Ragavan M. I. Thomas K. Medzhitova J. Brewer N. Goodman L. A. Bair-Merritt M. (2019). A systematic review of community-based research interventions for domestic violence survivors. Psychology of Violence, 9(2), 139–155. 10.1037/vio0000183

[bibr30-15248399211031540] Secret M. Abell M. L. Berlin T. (2011). The promise and challenge of practice-research collaborations: Guiding principles and strategies for initiating, designing, and implementing program evaluation research. Social Work, 56(1), 9–20. 10.1093/sw/56.1.9 21314067

[bibr31-15248399211031540] Serrata J. V. Macias R. L. Rosales A. Hernandez-Martinez M. Rodriguez R. Perilla J. L. (2017). Expanding evidence-based practice models for domestic violence initiatives: A community-centered approach. Psychology of Violence, 7(1), 158–165. 10.1037/vio0000051

[bibr32-15248399211031540] Trevisan M. S. Walser T. M. (2014). Evaluability assessment: Improving evaluation quality and use. Sage.

[bibr33-15248399211031540] Wight D. Wimbush E. Jepson R. Doi L. (2016). Six steps in quality intervention development (6SQuID). Journal of Epidemiology & Community Health, 70(5), 520–525. 10.1136/jech-2015-205952 26573236 PMC4853546

